# Emotional susceptibility trait modulates insula responses and functional connectivity in flavor processing

**DOI:** 10.3389/fnbeh.2015.00297

**Published:** 2015-11-05

**Authors:** Sjoerd J. H. Ebisch, Annalisa Bello, Grazia F. Spitoni, Mauro G. Perrucci, Vittorio Gallese, Giorgia Committeri, Concetta Pastorelli, Luigi Pizzamiglio

**Affiliations:** ^1^Department of Neuroscience, Imaging and Clinical Sciences, G. d'Annunzio University of Chieti-PescaraChieti, Italy; ^2^Institute of Advanced Biomedical Technologies, G. d'Annunzio University of Chieti-PescaraChieti, Italy; ^3^Department of Psychology, Sapienza UniversityRome, Italy; ^4^Laboratory of Neuropsychology, IRCCS Foundation Santa LuciaRome, Italy; ^5^Section of Physiology, Department of Neuroscience, Parma UniversityParma, Italy

**Keywords:** personality traits, emotional susceptibility, insula, cerebellum, taste, gustation, emotion, individual differences

## Abstract

The present study aimed at investigating the relationship between Emotional Susceptibility (ES), an aspect of the personality trait Neuroticism, and individual differences in the neural responses in anterior insula to primary sensory stimuli colored by affective valence, i.e., distasting or pleasantly tasting oral stimuli. In addition, it was studied whether intrinsic functional connectivity patterns of brain regions characterized by such differential responses could be related to ES. To this purpose 25 female participants underwent functional magnetic resonance imaging scanning, while being involved in a flavor experiment. During the experiment, flavor stimuli were administered consisting of small amounts of liquid with a different affective valence: neutral, pleasant, unpleasant. The results showed that individual differences in ES trait predicted distinct neural activity patterns to the different stimulus conditions in a region of left anterior insula that a previous meta-analysis revealed to be linked with olfacto-gustatory processing. Specifically, low ES was associated with enhanced neural responses to both pleasant and unpleasant stimuli, compared to neutral stimuli. By contrast, high ES participants showed equally strong neural responses to all types of stimuli without differentiating between the neutral and affective stimuli. Finally, during a task-free state, high ES trait appeared also to be related to decreased intrinsic functional connectivity between left anterior insula and left cerebellum. Our findings show that individual differences in ES are associated with differential anterior insula responses to primary sensory (flavor) stimuli as well as to intrinsic functional cortico-cerebellar connectivity, the latter suggesting a basis in the brain intrinsic functional architecture of the regulation of emotional experiences.

## Introduction

Since the beginning of this century, neuroscience increasingly devoted attention to the relationship between personality traits and the neural processing of affect (Canli and Amin, 2002; Hamann and Canli, 2004; Servaas et al., 2013). One of the main interests of this research is to link brain function with human temperamental and personality traits (Kennis et al., 2013). Generally, traits can be described as self-consistent manners of dealing with the environment. Thus, a key question in neuroscience is whether such behavioral consistency is associated with anatomical or functional brain properties characterizing individual personality.

Personality traits have been derived from the Five Factor Model of Personality (Costa and McCrae, 1992) or other personality theories that received a wide consensus (Gray and McNaughton, 2000; McNaughton and Corr, 2004). For instance, the Big Five Model (Costa and McCrae, 1990) categorizes personality traits in five dimensions or domains, comprising Extroversion, Neuroticism, Openness to experience, Agreeableness, and Conscientiousness (Digman, 1990). Of particular relevance for individual differences in reactions to emotional stimuli, Emotional Susceptibility (ES) has been proposed as a lower order personality trait related to the neuroticism dimension (Caprara and Pastorelli, 1989; Caprara et al., 1994). Neuroticism is characterized by anxiety, fear, moodiness, worry, envy, frustration, jealousy, and loneliness, whereas ES trait can be defined as the tendency to feel vulnerable, helpless, and inadequate in reaction to emotional stimuli (Caprara et al., 1985). According to Allport (1961), individual differences in the reactivity to emotional stimuli are a characteristic phenomenon of an individual's emotional nature and individual makeup which might be largely hereditary (see also Rothbart and Bates, 2006). Thus, although broad personality traits described by the Five Factor Model are suitable for investigating the neural basis of individual differences, lower-order traits of personality such as ES may augment specificity in the study of the relationship between personality traits and emotion processing (Caprara and Pastorelli, 1989; Caspi et al., 2005).

In a previous neuroimaging study, Iaria et al. (2008) found that two groups of healthy adults, selected as high and low in the ES trait, differed in their neural responses of the anterior insula to pictures with positive or negative emotional valence: only high ES participants bilaterally activated the anterior insula in response to visual images with affective valence. However, the response to visually presented affective stimuli requires a cognitive evaluation of what is seen and a reference to previous experience in order to attribute emotional meaning. Individual differences in the neural responses could therefore be based on higher-level cognitive processes related to the past social and psychological history of the participant.

Critical to be clarified remains the issue whether the relationship between personality traits and the neural processing of emotional stimuli involves phylogenetically older affective circuits or recently evolved cognitive systems. For example, in emotion processing, object qualities are processed through sensory pathways, while simultaneous affective processing in the limbic system can influence sensory processing by back projections (LeDoux, 1989). A concrete question would be whether activity in the anterior insula is modulated by ES only when the stimuli require an experiential evaluation. Alternatively, such modulation can also be found for primary sensory stimuli depending on evolutionarily ancient affective systems.

Taste or gustation is one of the primary senses that can evoke basic emotions (Chapman and Anderson, 2012; Rolls, 2015). For instance, oral distaste has been described as a primitive form of disgust representing an unpleasant emotion for the surveillance of body integrity, being evoked by substances that orally enter the body (Chapman et al., 2009). As such, it is central to elementary avoidance mechanisms evolved to prevent contamination by toxic or harmful food. In contrast, its positive counterpart, pleasant taste, can stimulate approaching behavior for nourishment. Relevant to be noted, taste perception, in its strict sense referring to the perception of sweetness, sourness, saltiness, bitterness, and umami, is intricately linked with other primary senses including olfaction and the trigeminal senses (de Araujo et al., 2003; Smith, 2012). Indeed, pure taste experiences are rare in everyday life characterized first and foremost by a combination of these basic senses to efficiently perceive flavors and attribute affective valence (Smith, 2012). Hence, the relationship between personality traits and the processing of oral stimuli with emotional valence could inform about how individual differences can originate in primary bodily experiences. In the present study the ES trait will be used in combination with neuro-functional measures during flavor experiences colored with emotional valence.

Previous neuroscientific research dedicated considerable attention to the negative emotion of disgust and most consistently highlighted anterior insula as part of the neural substrate for the experience of disgust (Murphy et al., 2003) and individual differences in this experience (Stark et al., 2007; Borg et al., 2013). Interestingly, anterior insula also was associated with the recognition of facial expressions of disgust based on lesion and fMRI data (Calder et al., 2000; Adolphs et al., 2003; Wicker et al., 2003). In addition, disgust-related behavior was induced by intracortical microstimulation of the monkey anterior insula (Caruana et al., 2011). However, most studies used pictures of disgusting events or facial expressions of disgust (Kennis et al., 2013). Few exceptions to this approach can be found introducing taste stimuli. These showed that taste and its affective coding modulates neural activity in anterior insula, also known as the putative primary taste cortex (Small et al., 1999; O'Doherty et al., 2001; de Araujo et al., 2003; Jabbi et al., 2007, 2008; Veldhuizen et al., 2011a; Rolls, 2015). A causal role of anterior insula in taste processing was suggested by lesion studies (Pritchard et al., 1999; see for a review, Small, 2010). Finally, anterior insula further has been identified by a recent meta-analysis as part of an olfacto-gustatory system (Kurth et al., 2010) where taste and olfaction information may converge for the subjective and affective processing of flavors (de Araujo et al., 2003; van den Bosch et al., 2014). However, although specific parts of anterior insula have been consistently associated with taste and flavor processing as well as related emotions (e.g., primary taste cortex), no relationship was examined between the affective coding of taste or flavor in the insular cortex and personality traits such as ES or neuroticism, yet.

The present study aimed to investigate whether ES trait is associated with differences in the anterior insula responses to flavor perception. For this purpose, functional magnetic resonance imaging was performed while negative (unpleasant), positive (pleasant), and neutral oral stimuli were administered in the mouth of healthy adult participants characterized by low of high ES trait. It is hypothesized that individuals with high scores on trait ES should show stronger activity in anterior insula to flavor stimuli, compared to low ES participants. Moreover, since brain function is not only to be explained in terms of focal neural activity, but also by communication within neural circuits, intrinsic functional connectivity of anterior insula was evaluated in relation to ES. A different pattern of insula intrinsic functional connectivity is expected between high and low ES participants, possibly contributing to individual differences in the regulation of affective responses, typically reduced in individuals with high ES trait (Caprara et al., 1983, 1987).

## Materials and methods

### Participants

Participants included in the fMRI experiment were selected from an original sample of 150 female university students (mean age = 21.27 years, standard deviation = 3.09, minimum = 18 years, maximum = 46 years) of the G. d'Annunzio University of Chieti. This study was carried out in accordance with the Declaration of Helsinki and approved by the local Ethics Committee, “Comitato Etico dell'Università degli Studi G. d'Annunzio e della ASL N. 2 Lanciano-Vasto-Chieti.” All participants gave their written informed consent before participating in this study. Recruitment was restricted to females (Veldhuizen et al., 2011b).

All participants in the original sample completed the Emotion Susceptibility Scale (ES) (Caprara et al., 1985). The ES is a 40-item self-report questionnaire based on a Likert scale with 6 response alternatives for each item (1, false; 6, true). An item example is: “*When I am afraid I totally loose control*.” Participants with an ES score below the 25th percentile were identified as “low ES,” whereas participants with a score above the 75th percentile were identified as “high ES.” Subsequently, 25 neurologically and psychiatrically healthy, right handed (Edinburgh Handedness Inventory) (Oldfield, 1971) participants were selected, out of which 13 participants with low ES (mean ES score = 3.27, standard deviation = 0.50) and 12 participants with high ES (mean ES score = 5.02, standard deviation = 0.64). Given that neuroticism/emotional stability has strong links with psychiatric disorders, in particular major depression in women (Kendler et al., 1993), and ES, it was important to exclude participants with clinical psychiatric disorders as a potentially confounding factor for interpreting group differences. The groups did not differ (*p* > 0.05) regarding age (low ES: mean age = 20.55 years, standard deviation = 1.37, minimum = 19, maximum = 24; high ES: mean age = 20.82, standard deviation = 0.63, minimum = 20, maximum = 22).

In addition to the ES scale, all participants completed the BFQ (Big Five Questionnaire; Caprara et al., 1993). The low ES and the high ES group significantly differed (*p* = 0.005) on the Emotional Stability dimension (low ES: mean score = 3.27, standard deviation = 0.90; high ES: mean score = 2.27, standard deviation = 0.61), but not in Openness, Conscientiousness, Extraversion or Agreeableness (all *p* > 0.05).

### Stimuli and experimental procedure

Flavor stimuli consisted of small amounts of liquid with different affective valence constituting the three experimental conditions: neutral (water), pleasant, or unpleasant (distaste). For the unpleasant stimuli, quinine (stock solution = 1.0 × 10^−3^ M) was diluted in 25 ml physiological solution and 250 ml water (e.g., Jabbi et al., 2007) together with 25 ml artichoke essence. This solution is perceived as bitter and induces an unpleasant sensation of distaste. For the pleasant stimuli, apple nectar was diluted in water characterized by a pleasant, sweet flavor. The neutral taste consisted in pure natural water.

The flavor stimuli were validated concerning their valence in a preliminary pilot study involving 15 participants (9 females; mean age = 24.4, standard deviation = 3.68) not participating in the fMRI experiment. During the pilot study, participants were asked to rate the intensity and pleasantness/unpleasantness of four different solutions obtained from the different concentration of quinine and artichoke serum diluted in water (unpleasant) and of four other solutions consisting of apple juice diluted in water (pleasant). Participants received the solutions while seated in a chair. After each sip the experimenter asked them to judge the valence of the stimulus as a forced dichotomous choice (“Is the liquid pleasant or unpleasant?”) and to rate its intensity on a Visual Analog Scale (VAS, 200 mm vertical line; “Indicate the intensity of the flavor by drawing a mark on the vertical bar with the lower end representing the minimum and the upper end the maximum intensity”). The eight solutions were randomly administered in two blocks of 24 stimuli each. Each block consisted of a randomized series of eight pleasant stimuli, eight unpleasant stimuli, and eight neutral stimuli (i.e., each solution was presented twice in each block). All participants (100%) judged the solutions 5, 6, 7, and 8 as unpleasant. Solutions 1, 2, and 3 were considered pleasant by all participants (100%), whereas, differently from our expectations, solution 4 was evaluated too sweet and unpleasant. We selected solution 3 (pleasant) and solution 8 (unpleasant) as the stimuli to use in the fMRI study, having unambiguous ratings regarding valence as well as having the highest intensity ratings, while not significantly differing in intensity ratings (*p* > 0.05). The results of the pilot study are presented in Table 1.

**Table 1:** **Results of the pilot study**.

	**Pleasant solutions (1–4)**				**Unpleasant solutions (5–8)**			
	**1**	**2**	***3***	**4**	**5**	**6**	**7**	***8***
Mass concentration (ρ_*i*_ = g/L)	0.25/0.5	0.5/0.5	***0.75/0.5***	1.0/0.5	0.005/0.5	0.01/0.5	0.015/0.5	***0.02/0.5***
Intensity (VAS; mean in mm ± standard deviation)	54.4 ± 12.5	75.2 ± 27.9	***84.9** ± **21.7***	28.8 ± 17.4	73.8 ± 11.1	85.5 ± 37.2	86.9 ± 39.5	***93.4** ± **19.4***
Pleasantness rating	P	P	***P***	U	U	U	U	***U***

*P, pleasant; U, unpleasant. VAS, Visual Analog Scale. Artichoke serum was kept constant across the different unpleasant solutions. The selected solutions for the fMRI study are indicated in bold italics*.

**Figure 1:**
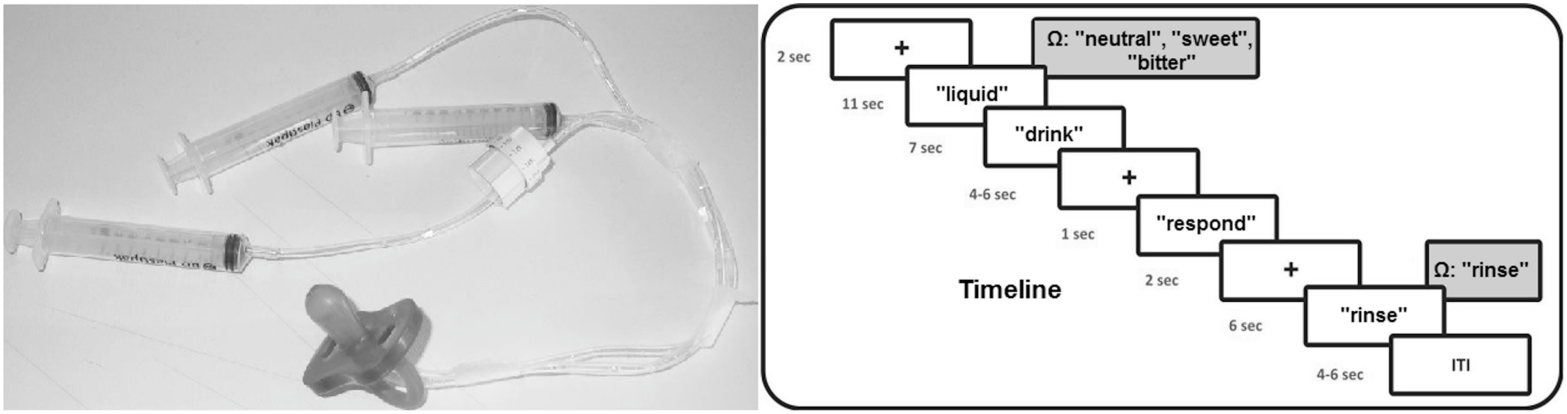
**Timeline of the experimental procedure and picture of the used materials for the liquid administration**. Ω represents the auditory cue received by the experimenter.

With respect to the fMRI experiment, after familiarization with the stimuli outside the scanner room, during fMRI scanning participants were invited to read and follow the instructions that were visually presented on a screen placed behind the MRI scanner. Participants could see the screen through a mirror placed above their eyes, while being in a supine position inside the scanner. The pleasant, unpleasant and neutral solutions were administered to the participants by using syringes (one for each experimental condition) connected to flexible tubes with a length of 2 m ending together in a pacifier (see Figure 1, left panel). Every trial, one of the three liquids (1 cc) was administered in participants' mouth by the experimenter. For this purpose, the experimenter who was standing next to the scanner was instructed by means of auditory cues presented through headphones: “neutral,” “sweet,” “bitter,” while the participant could not hear these cues. The experimenter was extensively trained before the start of the fMRI scanning sessions and the same experimenter (A.B.) administered the solutions to all participants.

Concerning the time course of the fMRI experiment, the experiment was executed in four fMRI blocks. Each block contained 12 flavor trials in random order (four neutral, four positive, and four negative). Thus, in total each condition was presented 16 times. Total fMRI scanning time for the flavor experimental blocks was approximately 31 min. Each trial started with a black fixation cross for 2 s after which a small amount of liquid was administered to participants, accompanied by the visual instruction “liquid” on the screen. After 11 s, the instruction “drink” appeared on the screen for 7 s requiring participants to swallow the liquid. Subsequently, a fixation cross was presented with a variable duration between 4 and 6 s followed by the instruction “respond” for 1 s, requiring participants to indicate by a right hand button press whether the liquid was perceived as unpleasant (index finger), neutral (middle finger), or pleasant (ring finger). After another fixation cross with a duration of 2 s, the instruction “rinse” was presented on the screen requiring to rinse the mouth for 6 s with 2 cc of water administered by the experimenter. Experimental trials were separated by a fixation cross with a duration varying between 4 and 6 s. Total trial duration was 39 s on average. Time courses of the experimental trials are visualized in Figure 1 (right panel).

### fMRI data acquisition

For each participant, blood oxygen level dependent (BOLD) contrast functional imaging was performed with a Philips Achieva scanner at 3T at the Institute of Advanced Biomedical Technologies (ITAB), G. d'Annunzio University, Chieti, Italy. Concerning the flavor experiment, T2^*^-weighted functional data were collected with an eight channel phased array head coil. EPI data (gradient echo pulse sequence) were acquired from 40 slices with a thickness of 3.59 mm (in-plane voxel size 3.594 × 3.594 mm, TR = 2000 ms, TE = 64 ms, SENSE factor = 2, flip angle = 80°, Field of View = 230 mm). In addition, two task-free, eyes-open (fixation cross) scanning blocks were performed consisting of 160 functional volumes each. Slices were oriented parallel to the AC–PC axis of the observer's brain. Finally, a T1-weighted anatomical (3D MPRAGE pulse sequence; 1 mm isotropic voxels) scanning block was performed.

### Task-evoked fMRI data preprocessing and analysis

Raw data were analyzed with Brain Voyager QX 2.3 software (Brain Innovation, Maastricht, The Netherlands). Due to T1 saturation effects, the first five scans of each run were discarded from the analysis. Preprocessing of functional data included slice scan time correction, motion correction and removal of linear trends from voxel time series. A three-dimensional motion correction was performed with a rigid-body transformation to match each functional volume to the reference volume estimating three translation and three rotation parameters. Scanning blocks with head movements larger than the size of one voxel were excluded from further analysis. Preprocessed functional volumes of a participant were co-registered with the corresponding structural data set. As the 2D functional and 3D structural measurements were acquired in the same session, the co-registration transformation was determined using the slice position parameters of the functional images and the position parameters of the structural volume. Structural and functional volumes were transformed into the Talairach space (Talairach and Tournoux, 1988) using a piecewise affine and continuous transformation. Functional volumes were resampled at a voxel size of 3 × 3 × 3 mm and spatially smoothed with a Gaussian kernel of 6 mm full-width half maximum to account for interindividual variability.

The fMRI blocks were modeled by means of a two gamma hemodynamic response function using predictors for the different flavor conditions: three regressors including “liquid” and “drink” phases, representing the flavor conditions (neutral, pleasant, or unpleasant), one regressor for the “respond” phase, and one regressor for the “rinse” phase. The fixation cross periods were defined as baseline period (rest) and not modeled by hemodynamic response function.

Prior to statistical analysis, percent signal change normalization of the time series from the different runs was performed. The parameters (beta values) estimated from individual participants analysis were entered in a second level voxel-wise random effect group analysis in order to search for activated areas that were consistent for the whole group of participants. Group statistical maps of neural activation and its modulation by experimental condition and group were calculated using a *p* < 0.001. This value together with an estimate of the spatial correlation of voxels were used as input in a Monte Carlo simulation (1000 simulations) to access the overall significance level and to determine a cluster size threshold (k) in order to obtain a significance level of *p* < 0.05 cluster level corrected for multiple comparisons (Forman et al., 1995).

Between-group statistical analysis focused on the three flavor conditions defined by the three regressors (pleasant, unpleasant, and neutral) that included both the “liquid” and the “drink” phase. In this period (from the 3rd until the 20th s of each trial) the liquid was present in the mouth of the participant. In order to focus on insula activity patterns during the experiment, especially insula regions related to disgust, a mask (total mask size = 22,626 voxels) was created based on the coordinates reported in a recent meta-analysis on insula function (Kurth et al., 2010). Specifically, a mask was created consisting of spherical voxel clusters with a radius of 9 mm centered on the coordinates related to the specific meta-analysis of the olfacto-gustatory system: (1) −35, 20, 5; (2) −36, 10, 11; (3) −33, 0, 11; (4) 44, 24, −5; (5) 44, 9, 2; (6) 40, 12, −6. These coordinates were transformed to the original MNI space in Talairach space by using the algorithm developed by Lancaster et al. (2007).

Voxel-wise analysis of variance (ANOVA) was performed within the disgust insula mask using flavor condition as within-participant factor (three levels: neutral, pleasant, unpleasant) and ES group as between-participant factor (two levels: high, low).

### Task-free fMRI data preprocessing and analysis

In addition to the fMRI preprocessing steps used for task-fMRI data, for intrinsic functional connectivity analysis, a second step of data preprocessing (Fox and Raichle, 2007; Van Dijk et al., 2010; Ebisch et al., 2011, 2014; Power et al., 2014) was performed by using self-devised MATLAB scripts (The Mathworks Inc., Natick, MA) including: (1) band pass filtering between 0.009 and 0.08 Hz; (2) regression of global, white matter, and ventricle signals, and their first derivatives; (3) regression of three dimensional motion parameters, and their first derivatives; (4) regression of task-related BOLD fluctuations; (5) scrubbing of motion affected functional volumes (FD threshold = 0.5%; DVARS threshold = 4.6%).

Functional connectivity is operationally defined as the statistical dependence between low-frequency (0.009–0.08 Hz) BOLD signals in distant brain regions (Fox and Raichle, 2007; Van Dijk et al., 2010). Functional connectivity analysis identifies temporally correlated patterns of ongoing activity across brain regions with direct or indirect anatomical connections, and is considered to represent an index of intrinsic long-range communication across the brain.

Seed-based analysis of intrinsic functional connectivity was performed identifying temporally correlated patterns of brain activity across brain voxels. Functional interaction maps were calculated by means of voxel-wise, whole brain analyses for the seed ROI defined as a sphere with a 6 mm radius. The seed ROI was functionally based on the peak coordinate of the activation cluster in left anterior insula (coordinates: *x* = −32; *y* = 11; *z* = 7) showing a significant group ^*^ condition interaction effect in the previously described task-fMRI analysis. After applying Fisher's r-to-z transformation to each correlation map, random-effect analysis was performed independently for each of the two ES groups in order to reveal functional connectivity patterns that were consistent across participants. Statistical significance was determined by means of one-sample *t*-tests. Group statistical maps were thresholded at *p* < 0.01 corrected for multiple comparisons by the False Discovery Rate (FDR). To test for significant differences between the low and high ES group, independent-sample *t*-tests between the functional connectivity maps were calculated for the left anterior insula seed. Statistical maps of these between-group contrasts were thresholded at *p* < 0.05 FDR corrected with a cluster size of *k* > 8.

## Results

### Behavioral results: Recognition of stimulus categories

Behavioral results (response errors and reaction times) are provided in Table 2. Due to technical problems, behavioral data (errors and reaction times) of one high ES participant were not recorded. ANOVA of response errors during fMRI scanning yielded a significant group (low ES, high ES) ^*^ experimental condition (neutral, pleasant, unpleasant) effect [*F*_(2, 44)_ = 4.068, *p* < 0.05] demonstrating differential response patterns in the low and high ES group. No main effect of group or of experimental condition was detected (both *p* > 0.1).

**Table 2:** **Behavioral data obtained during the fMRI experiment: number of stimulus category recognition errors and reaction times for the low and high ES group**.

	**# Errors (mean ± standard deviation)**			**Reaction time in ms (mean ± standard deviation)**		
	**Neutral**	**Positive**	**Negative**	**Neutral**	**Positive**	**Negative**
Low ES	1.15 ± 1.52	0.23 ± 0.44	0.15 ± 0.38	1062 ± 358	3264 ± 1470	3201 ± 1133
High ES	0.27 ± 0.65	0.36 ± 0.92	0.36 ± 0.92	1086 ± 421	2998 ± 1129	3239 ± 1316

*Post-hoc* contrasts showed a significant interaction of the between-participant group factor (low ES, high ES) with the within-participant contrast between the neutral and the pleasant condition [*F*_(1, 22)_ = 4.842, *p* < 0.04] and a trend toward significance for the contrast between the neutral and the unpleasant condition [*F*_(1, 22)_ = 4.009, *p* < 0.06]. When analyzing the groups separately, concerning the low ES group, a significant within-participant effect was detected [*F*_(2, 24)_ = 5.19, *p* = 0.01], with a significant difference in errors between the pleasant and the neutral condition [*F*_(1, 12)_ = 5.799, *p* = 0.03] as well as between the unpleasant and the neutral condition [*F*_(1, 12)_ = 5.2, *p* = 0.04]: average number or errors was higher for the neutral condition, compared to the pleasant and unpleasant conditions, in the low ES group. With respect to the high ES group, no significant within-participant effect could be detected regarding response errors (*p* > 0.05). Finally, comparing the errors of the two groups for the different experimental conditions, a trend toward a significant difference was found for the neutral condition [*F*_(1, 22)_ = 3.193, *p* < 0.09], but not for the pleasant and unpleasant stimuli (*p* > 0.4), due to a higher number of errors in the recognition of the neutral stimuli in the low ES group, compared to the high ES group. In other words, these data suggest that, while the two groups both categorized the pleasant and unpleasant stimuli rather unambiguously, the groups tended to judge the neutral stimuli differently.

Regarding reaction times, ANOVA neither showed a significant main effect of group nor a significant group ^*^ experimental main effect (*p* > 0.5). A significant main effect of experimental condition was found [*F*_(2, 44)_ = 95.54, *p* < 0.001], indicating increased reaction times for the pleasant [*F*_(1, 22)_ = 99.98, *p* < 0.001] as well as for the unpleasant condition [*F*_(1, 22)_ = 143.44, *p* < 0.001], compared to the neutral condition.

### Task-evoked fMRI results: Low ES group

Voxel-wise analysis based on *t*-tests and thresholded at *p* < 0.05 corrected [*p* < 0.001 uncorrected, *t*_(12)_ > 4.32, *k* > 8] of the low ES group showed increased activity (positive BOLD modulation) for the unpleasant condition, compared to baseline, in bilateral ventral postcentral gyrus (including gustatory cortex) extending to precentral gyrus (premotor cortex), bilateral middle frontal gyrus (lateral prefrontal cortex), bilateral medial frontal gyrus (supplementary motor cortex), and right mid insula. Decreased activity (negative BOLD modulation), compared to baseline, was detected in bilateral medial frontal gyrus (medial prefrontal cortex), bilateral ventral posterior cingulate gyrus, bilateral parahippocampal gyrus, bilateral middle temporal gyrus/angular gyrus, and bilateral lingual gyrus/inferior occipital gyrus/middle occipital gyrus.

Concerning the pleasant condition increased activity, compared to baseline, was found in bilateral postcentral/precentral gyrus (including gustatory and premotor cortex), bilateral medial frontal gyrus (supplementary motor cortex), bilateral mid/posterior insula, bilateral cerebellum, bilateral supramarginal gyrus, and right middle frontal gyrus (lateral prefrontal cortex). Decreased activity, compared to baseline, was detected in bilateral ventral posterior cingulate cortex, and bilateral lingual/inferior occipital/middle occipital/fusiform gyrus.

For the neutral condition, increased activity, compared to baseline, was shown in bilateral supramarginal gyrus, bilateral ventral postcentral/precentral gyrus (including gustatory and premotor cortex), bilateral middle/inferior frontal gyrus (lateral prefrontal cortex), bilateral cerebellum, right ventral postcentral gyrus. Decreased activity, compared to baseline, was found in bilateral medial frontal gyrus (medial prefrontal cortex), bilateral anterior, mid and posterior cingulate cortex, bilateral parahippocampal/fusiform gyrus, bilateral lingual/inferior occipital/middle occipital gyrus, and bilateral anterior middle temporal gyrus.

The voxel-wise ANOVA thresholded at *p* < 0.05 corrected [*p* < 0.001 uncorrected, *F*_(2, 24)_ > 9.32, *k* > 8; see Table 3] yielded a significant main effect of experimental condition in the low ES group in bilateral superior/transverse temporal gyrus, bilateral angular/inferior parietal gyrus (posterior parietal cortex), right anterior and posterior parahippocampal gyrus, right ventral mid insula, and left superior occipital gyrus (extrastriate cortex).

**Table 3:** **Anatomical and statistical details regarding modulation of neural activity by experimental condition (i.e., differentiating between neutral, pleasant, unpleasant stimuli) in flavor processing in the low and high ES group**.

**Brain region**	**Talairach coordinates (x, y, z)**	**Cluster size**	**Peak *F*-value**	**Peak *p*-value**
**LOW ES GROUP**				
Right posterior parietal cortex	33, −54, 36	1944	17.44	0.000021
Left posterior parietal cortex	−33, −61, 38	270	12.19	0.000221
Right superior temporal gyrus	45, −30, 8	891	17.48	0.000021
Left superior temporal gyrus	−54, −31, 16	378	14.29	0.000082
Right posterior parahippocampal gyrus	15, −39, −5	2916	19.33	0.000010
Right anterior parahippocampal gyrus	17, 0, −11	486	15.10	0.000057
Right ventral mid insula	37, −3, −9	432	11.11	0.000384
Left extrastriate cortex	−35, −82, 33	540	15.90	0.000040
**HIGH ES GROUP**				
Right inferior frontal gyrus	47, 30, 6	1890	20.87	0.000008
Left Cerebellum	−15, −52, −19	4266	18.61	0.000019
Left middle frontal gyrus	−56, 18, 34	2619	22.70	0.000004
Left precentral gyrus	−37, −1, 39	432	20.69	0.000009
Right anterior insula	39, 6, 6	999	20.98	0.000008
Right ventral precentral gyrus	61, −9, 35	837	29.63	0.000001
Right middle occipital gyrus	28, −83, 21	297	14.99	0.000078
Right middle temporal gyrus	58, −21, −14	621	13.73	0.000135
Right posterior parietal cortex	28, −76, 38	864	14.33	0.000104
Left posterior parietal cortex	−30, −70, 42	945	17.68	0.000026

### Task-evoked fMRI results: High ES group

Voxel-wise analysis based on *t*-tests and thresholded at *p* < 0.05 corrected [*p* < 0.001 uncorrected, *t*_(11)_ > 4.44, *k* > 8] of the high ES group showed increased activity (positive BOLD modulation) for the unpleasant condition, compared to baseline, in bilateral anterior/mid/posterior insula, bilateral postcentral/precentral gyrus (including gustatory cortex), bilateral medial frontal gyrus (supplementary motor cortex), bilateral dorsal anterior cingulate gyrus, bilateral cerebellum, bilateral middle temporal/occipital gyrus, right middle frontal gyrus (lateral prefrontal cortex), and right putamen. Decreased activity (negative BOLD modulation), compared to baseline, was detected in bilateral subgenual anterior cingulate gyrus, and bilateral lingual/inferior occipital/middle occipital gyrus.

Concerning the pleasant condition increased activity, compared to baseline, was found in bilateral postcentral/precentral gyrus (including gustatory cortex), bilateral cerebellum, right middle frontal gyrus (lateral prefrontal cortex), and right anterior/mid/posterior insula. Decreased activity, compared to baseline, was detected in bilateral medial frontal gyrus (medial prefrontal cortex), bilateral ventral posterior cingulate cortex/precuneus, bilateral posterior middle temporal gyrus, and right lingual/inferior occipital/middle occipital gyrus.

For the neutral condition, increased activity, compared to baseline, was shown in bilateral anterior insula, bilateral postcentral/precentral gyrus (including gustatory and premotor cortex), bilateral medial/superior frontal gyrus (supplementary motor cortex), bilateral cerebellum, bilateral supramarginal gyrus, bilateral inferior parietal lobule, bilateral intraparietal sulcus, right middle frontal gyrus (lateral prefrontal cortex), and left dorsal anterior cingulate cortex. Decreased activity, compared to baseline, was found in bilateral medial frontal gyrus (medial prefrontal cortex), bilateral ventral posterior cingulate cortex/precuneus.

The voxel-wise ANOVA thresholded at *p* < 0.05 corrected [*p* < 0.001 uncorrected, *F*_(2, 22)_ > 9.61, *k* > 8; see Table 3] yielded a significant main effect of experimental condition in the high ES group in bilateral posterior parietal cortex, right inferior frontal gyrus, cerebellum, left middle frontal gyrus (lateral prefrontal cortex), left precentral gyrus, right anterior insula, right ventral precentral gyrus, right middle occipital gyrus, and right mid middle temporal gyrus.

### Task-evoked fMRI results: Low ES vs. high ES group

Voxel-wise ANOVA (one within-participant factor and one between-participant factor) thresholded at *p* < 0.05 corrected [*p* < 0.001 uncorrected, *F*_(2, 46)_ > 8.06, *k* > 5] yielded a significant within-participant effect of experimental condition (neutral, pleasant, unpleasant) in right anterior insula, whereas no significant between-participant effect of group (low ES, high ES) could be detected within the insula disgust mask [*p* < 0.001 uncorrected, *F*_(1, 23)_ > 14.20, *k* > 5]. A significant condition ^*^ group interaction effect was found in left anterior insula [*p* < 0.001 uncorrected, *F*_(2, 46)_ > 10.44]. Observed power of the ANOVA as an estimate of the power based on the observed effect size was 0.94, and the groups did not differ regarding the variance of their neural responses to the stimuli (*p* > 0.05).

To provide more insight in this interaction effect, *post-hoc* analyses were performed on the individual beta values calculated from the average signal time course of all the voxels included the activation clusters obtained by the voxel-wise analysis. *Post-hoc* contrasts showed a significant interaction of the between-participant group factor (low ES, high ES) with the within-participant contrasts between the neutral and the pleasant [*F*_(2, 46)_ = 11.525, *p* = 0.002] and between the neutral and the unpleasant [*F*_(1, 23)_ = 4.728, *p* = 0.04] condition. When analyzing the groups separately, concerning the low ES group, a significant within-participant effect was detected [*F*_(2, 24)_ = 5.680, *p* = 0.02], with a significant difference between the pleasant and the neutral condition [*F*_(1, 12)_ = 6.246, *p* = 0.03] as well as between the unpleasant and the neutral condition [*F*_(1, 12)_ = 7.314, *p* = 0.02]: average beta-value in anterior insula was higher for the pleasant and unpleasant conditions, compared to the neutral condition, in the low ES group. With respect to the high ES group, no significant within-participant effect could be detected in left anterior insula (*p* > 0.05). Finally, comparing the two groups for the different experimental conditions, a significant difference was found for the neutral condition [*F*_(1, 25)_ = 11.392, *p* = 0.003], indicating a stronger activation for the neutral stimulus in the high ES group, compared to the low ES group. No differences could be detected between the groups for the pleasant or the unpleasant condition (*p* > 0.05).

**Figure 2:**
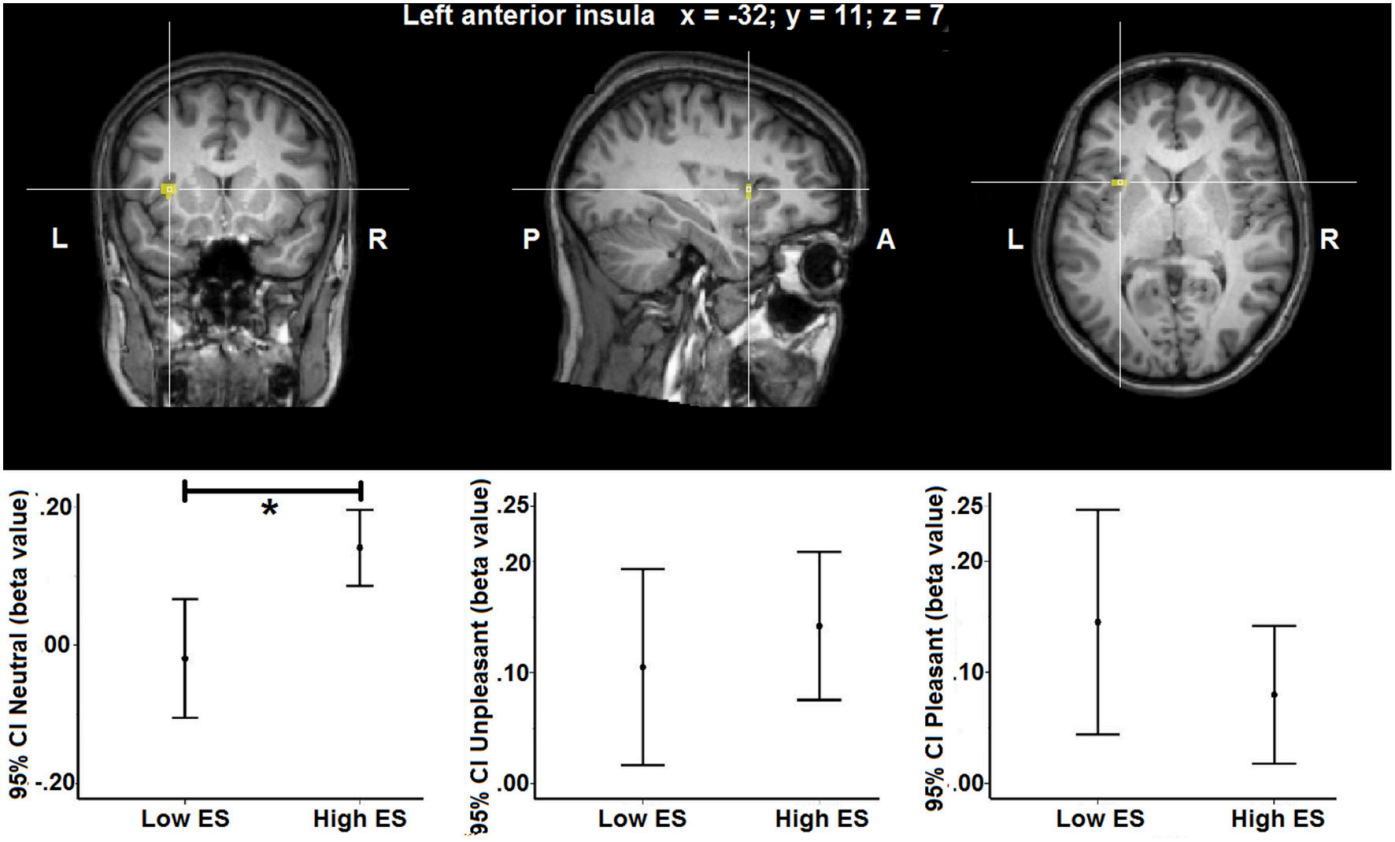
**Group statistical maps of the group (low ES, high ES) × experimental condition (unpleasant, pleasant, neutral) interaction effect showing a statistically significant cluster in left anterior insula [***F***_(2, 46)_ > 10.44, ***p*** > 0.05 corrected]**. Graphs show 95% Confidence Intervals (CI) of the beta values for each condition separated by ES group, compared to baseline. ^*^significant effect of contrast at *p* < 0.05 based on *post-hoc* analysis.

Group statistical maps and graphs regarding the interaction effect in left anterior insula are shown in Figure 2. Graphs provide mean beta-values and 95% Confidence Intervals (CI), the latter reflecting the estimated range of values that contains the true mean of the population with a 95% likelihood. Although sample size of the groups was relatively small, the 95% CI indicates relatively narrow ranges of values as well as no overlap between the low and high ES group for the neutral condition. This suggests accurate estimations and reliable differences in mean between the low and high ES in their responses to the neutral stimulus. More detailed information about localization and statistical effects are provided in Table 4.

**Table 4:** **Anatomical and statistical details regarding modulation of left anterior insula activity in flavor processing by ES**.

**Brain region**	**Talairach coordinates (x, y, z)**	**Cluster size**	**Group**	**Beta value (± SE) for each condition**			**Peak *F*-value (group × condition interaction)**	***p*-value (group × condition interaction)**
				**Unpleasant**	**Pleasant**	**Neutral**		
Left anterior insula	−32, 11, 7	432	Low ES	0.10 (± 0.04)	0.15 (± 0.05)	−0.02 (± 0.04)	10.44	<0.001
			High ES	0.14 (± 0.03)	0.08 (± 0.03)	0.14 (± 0.03)		

A whole-brain, voxel-wise analysis confirmed a significant interaction effect in the same left anterior insula cluster, and did not yield additional effects in other insular regions [*p* < 0.001 uncorrected, *F*_(1, 23)_ > 14.20, *k* > 5].

### Task-free fMRI results

In the low ES group, intrinsic functional connectivity maps of the left anterior insula seed ROI thresholded at *p* < 0.05 corrected (*t* > 4.22) showed significant connectivity with the bilateral medial frontal gyrus (supplementary motor area), bilateral dorsal anterior cingulate cortex, bilateral ventral precentral gyrus, bilateral postcentral gyrus, bilateral middle frontal gyrus (lateral prefrontal cortex), bilateral supramarginal gyrus, bilateral anterior/mid insula, right inferior parietal lobule, right putamen, right cerebellum, left posterior insula, and left posterior middle temporal gyrus.

In the high ES group, intrinsic functional connectivity maps of the left anterior insula seed ROI thresholded at *p* < 0.05 corrected (*t* > 4.53) showed significant connectivity with the bilateral dorsal anterior cingulate cortex, bilateral supramarginal gyrus, bilateral ventral postcentral gyrus, bilateral ventral precentral gyrus, bilateral middle/inferior frontal gyrus (lateral prefrontal cortex), bilateral anterior/mid insula, bilateral putamen, and left inferior parietal lobule.

**Figure 3:**
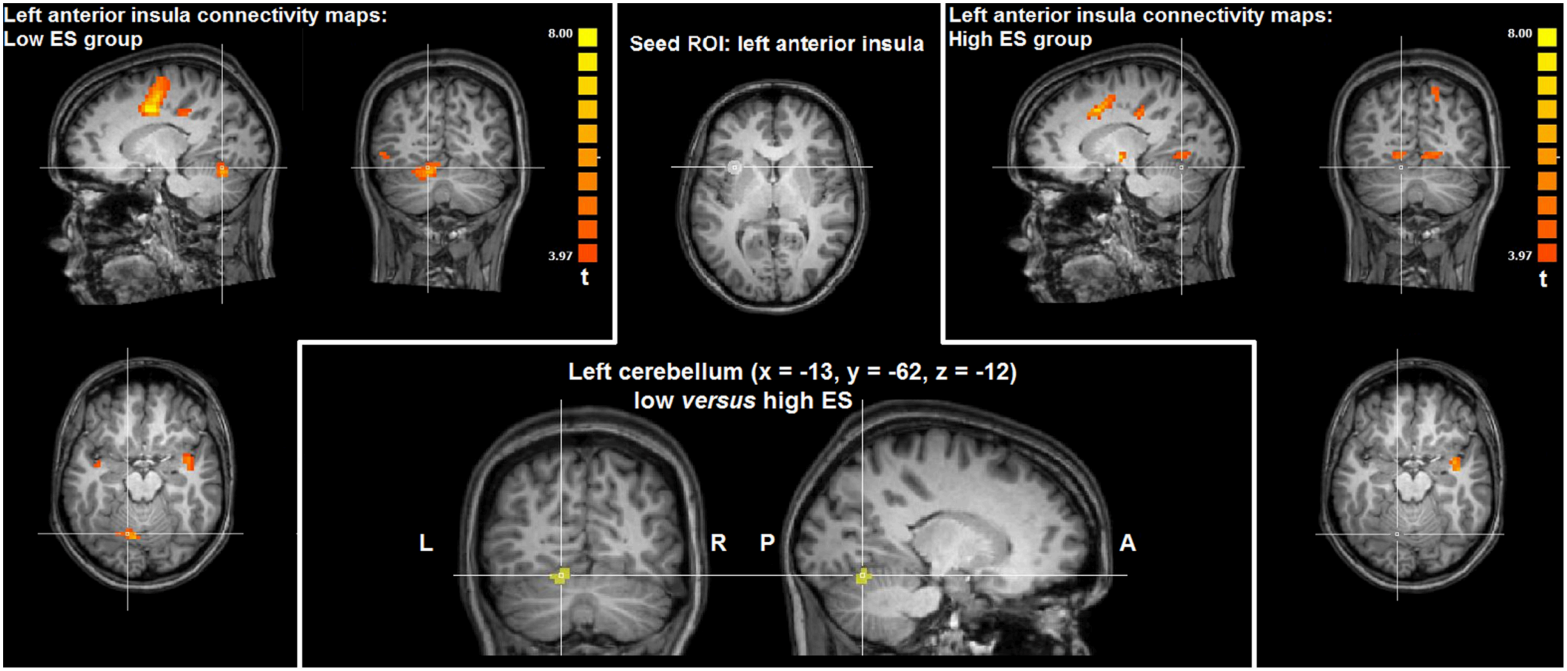
**Group statistical maps of intrinsic functional connectivity analysis based on the left anterior insula seed ROI showing significantly higher connectivity (***t*** > 3.20, ***p*** < 0.05 corrected) in left cerebellum in the low ES group, compared to the high ES group**.

Directly comparing the group statistical maps of left anterior insula connectivity of the low and high ES groups (*t* > 3.20, *p* < 0.05 corrected) yielded higher functional connectivity in left cerebellum (paravermal lobule IV) in the low ES group, compared to the high ES group (*x* = −13, *y* = −62, *z* = −12; cluster size = 432 voxels). Results in left cerebellum obtained by intrinsic functional connectivity analysis are visualized in Figure 3.

## Discussion

The present study aimed at investigating the modulation of neural activity during flavor experiences and functional connectivity of insula by individual differences in ES trait. The results showed differential neural activity patterns in left anterior insula when comparing low and high ES participants. Whereas the low ES group showed an enhanced neural response to both the pleasant stimulus and the unpleasant stimulus, compared to the neutral stimulus, the high ES group did not exhibit this differentiation between the experimental conditions. The high ES group, instead, activated left anterior insula during the neutral stimulus with a similar intensity as during the pleasant and unpleasant stimuli, and with a significantly higher intensity, compared to the low ES group.

With respect to the functional properties of anterior insula, it has been proposed that it is involved in attending interoceptive stimuli (Critchley, 2005), emotional feelings (Craig, 2002; Critchley, 2005), and self-awareness (Craig, 2009). Furthermore, others argued that anterior insula also more generally contributes to the encoding of external stimuli for subsequent processing (Menon and Uddin, 2010), and functional integration between systems (Augustine, 1996; Kurth et al., 2010). Finally, and more specifically related to the present experiment, a large amount of studies suggested that anterior insula accommodates a primary taste cortex coding taste quality (Frey and Petrides, 1999; Pritchard et al., 1999; Scott and Plata-Salaman, 1999; Small et al., 1999; Zald and Pardo, 2000; O'Doherty et al., 2001; Katz et al., 2002; Accolla et al., 2007; Small, 2010; Veldhuizen et al., 2011a; Rolls, 2015), as well as an olfacto-gustation system (Kurth et al., 2010) at the basis of flavor perception due to the convergence of gustatory and olfactory information (de Araujo et al., 2003). Indeed, anterior insula lesions, sometimes together with basal ganglia, have been reported to cause deficits in the recognition and experience of disgust (Calder et al., 2000; Adolphs et al., 2003) suggesting an insula-basal ganglia system at the basis of the processing of disgust (Calder et al., 2001; Calder, 2003).

The reported effects were localized in anterior insula regions associated with olfacto-gustatory processing by previous fMRI studies as suggested by a recent meta-analysis (Kurth et al., 2010). This implies that among insula regions involved in taste processing, likely in combination with olfactory processing, especially left anterior insula is modulated by ES trait. This possibly contributes to individual differences in basic emotional experiences induced by oral stimuli. Moreover, the finding that modulation of left anterior insula by ES was found for primary sensory stimuli depending on an evolutionarily ancient olfacto-gustatory system (Chapman and Anderson, 2012; Rolls, 2015), implies that the relationship between personality traits and the neural processing of emotional stimuli may not necessarily be confined to higher cognitive systems, but could include phylogenetically older affective circuits, too.

Previous studies on personality traits in the disgust domain focused on the relationship between affective visual stimuli and individual disgust sensitivity. Individual disgust sensitivity constitutes a general tendency to respond with a transitory disgust state not only to distasting stimuli, but also to an ample variety of environmental stimuli perceived as repulsive (Schienle et al., 2005; Mataix-Cols et al., 2008), and significantly correlates with neuroticism (Haidt and McCauley, 1994), anxiousness (Cisler et al., 2009), and sensibility to somatic symptomatology of anxiousness (Cisler et al., 2007). The present results are partially in line with this previous work that showed modulation of neural activity in brain circuits involved in disgust processing, like left anterior insula, by personality traits, in particular disgust sensitivity (Borg et al., 2013). Furthermore, Stark et al. (2007) found bilaterally increased insular activity by visual stimuli inducing disgust, which correlated with participants' ratings of experienced disgust. Finally, significant correlations were found between morphometric measures of anterior insula and individual differences in disgust propensity (Scharmuller and Schienle, 2012). Our findings essentially add to this research by combining the neural processing of flavor stimuli (pleasant as well as unpleasant or disgusting) in anterior insula with measures of personality traits ES, that is, explaining individual differences in dealing with primary sensory stimuli with affective valence.

Nevertheless, some relevant differences with existing literature need to be noted as well. Previous studies showed enhanced neural responses in anterior insula to emotional stimuli, including unpleasant or pleasant visual stimuli, linked with high disgust sensitivity (Borg et al., 2013) or ES (Iaria et al., 2008). Differently, in the present study, increased activity in high ES participants, compared to low SE participants, was detected for the neutral condition, but not for the affective conditions. In contrast to the low ES group, the high ES group rather appeared not to discriminate between the stimulus categories at the neuro-functional level.

A possible interpretation of this pattern could be based on the fact that during the fMRI experiment, participants received a random sequence of neutral and affective stimuli. This experimental context recalls something occurring in the visual perceptual domain, the “crowding effect” (Pelli et al., 2004; Pelli and Tillman, 2008). When a visual stimulus with clear physical properties (i.e., a printed letter of the alphabet) is shown on the screen, participants can easily recognize the stimulus. However, when the same physical stimulus is presented in contexts with an increasing number of distracting information, its discriminability decreases with the complexification of the visual pattern. In our case the continuous sequence of stimuli of different emotional value can play the role of crowding, decreasing the emotional differentiation in the high ES group. Thus, the similarity with the well-known “crowding effect” in vision can help to understand why the group with high ES, at variance with the low ES group, does not respond in a differential way to the three stimulus categories.

Alternatively, the activation patterns could reflect a tendency in the high ES group to automatically react in an emotional way also to the neutral stimuli, because these were mixed with the affective stimuli. To put it differently, every time a liquid was administered, the possibility (67%) existed for the participant that the stimulus had an affective valence. Considering this chance, anterior insula activity may be boosted as soon as “something” is administered in the mouth, before becoming consciously aware of the nature of the stimulus. A related effect was reported by Mazzola et al. (2013), showing a modulation of neutral visual stimulus processing (hand actions accompanied by neutral faces) when presented in distinct sequences of affective stimuli (hand actions accompanied by different facial expressions of emotion). Moreover, insular taste responses are also sensitive to expectation (Nitschke et al., 2006; Veldhuizen et al., 2011b). This would suggest an immediate and preliminary affective reaction in high ES participants as opposed to a more regulated, and differentiated, response in the low ES group.

Although the experimental paradigm used in the present study does not allow to distinguish between these alternative explanations of the results, they are not mutually exclusive and both explanations might clarify why people with high ES trait in everyday situations show difficulties in regulating properly their affective responses to events suddenly appearing in their environment in certain contexts. Since previous studies showed that taste processing in insula can be modulated by attention (Veldhuizen et al., 2007; Grabenhorst and Rolls, 2008; Bender et al., 2009), further studies are warranted to examine whether these individual differences are pre-attentive or can be mediated by attention. For instance, a recent study showed that the Attention Regulation facet of interoceptive awareness as multidimensionally assessed through the self-report MAIA (Mehling et al., 2012) is particularly relevant in explaining the variability of ES scores (Calì et al., 2015).

Of particular interest, the low and high ES group tended to differ in the explicit recognition (i.e., stimulus discrimination) specifically of the neutral stimulus category. In apparent contrast with the fMRI results, the low ES group tended to discriminate the neutral stimuli less from the affective stimuli; they showed a trend toward significance in rating the neutral stimulus more often as either pleasant or unpleasant. This may indicate that the groups also differed in their subjective perception of the neutral stimuli, while there were no differences in their subjective categorization of the affective stimuli. Accordingly, anterior insula also has been related to individual differences in flavor preferences (van den Bosch et al., 2014). However, given that the revealed neural activity patterns and the behavioral evaluation of the stimuli during fMRI scanning correlated in an opposite direction with ES trait, these data imply that the undifferentiated neural responses in the high ES group to flavor stimuli is independent of the cognitive recognition of the affective valence of these stimuli. The neural and behavioral responses could speculatively be linked with different stages of stimulus processing: while the former could reflect an initial automatic reaction to a sensory stimulus, the latter may concern a successive and more cognitive analysis of stimulus valence.

In addition to these task-related fMRI results, task-free fMRI data provided information about intrinsic functional connectivity patterns of the anterior insula cluster modulated by ES trait. Functional connectivity analysis showed that weaker functional connections of left anterior insula with cerebellum (paravermal lobule IV) could be linked with high ES. In recent years, a considerable amount of evidence has been provided concerning the role of the cerebellum in modulating responses to affective stimuli (Moulton et al., 2011), including disgust associated with paravermal lobule IV activation (Baumann and Mattingley, 2012). Indeed, the cerebellum may constitute an important node within a cortico-limbic network centered on dorsal anterior cingulate cortex and anterior insula involved in the detection, integration, and filtering of emotional information (Seeley et al., 2007). Furthermore, structural differences in the cerebellar cortex and its white matter connections associated with personality differences was described (Laricchiuta et al., 2014; Petrosini et al., 2015). Finally, clinical studies reported that deficits in affective regulation related to the cognitive-affective cerebellar syndrome are worse when lesions involve the cerebellar vermis and paravermis (Schmahmann, 1991).

Hence, we propose that functional connections between anterior insula and cerebellum could be involved in emotion regulation when encountering affective stimuli. Given that functional connectivity in terms of a statistical dependence between ongoing low-frequency BOLD signals in distant brain regions can be considered an index of the intrinsic functional organization of the brain (Fox and Raichle, 2007; Van Dijk et al., 2010), weaker connectivity between insula and cerebellum could contribute to a neural predisposition for a reduced regulation of emotional responses as typically observed in high ES individuals (Caprara et al., 1983, 1987).

Some final issues need to be mentioned. Firstly, although the present study focused on flavor perception in the context of previous neuroimaging work on taste or gustatory processing, especially with emotional valence including unpleasant (disgusting) and pleasant taste, artichoke serum was added to the unpleasant stimulus and apple nectar was used for the pleasant stimulus to emphasize their experience in terms of either pleasant or unpleasant, rather than sweet and bitter. Therefore, the stimuli may not have been purely gustatory, but likely involved additional olfactory processing as well, allowing the perception of flavor as a more complex quality. However, we think that this is not a confounding factor for the interpretation of our results for several reasons. Flavor is a multisensory experience composed of two basic senses, taste and olfaction, with additional contributions of the trigeminal senses. This suggests that flavor perception nevertheless is based on primary sensory-affective mechanisms. In addition, certain anterior insula regions have been identified as part of an integral olfacto-gustatory system contributing to both gustatory and olfactory processing (de Araujo et al., 2003; Kurth et al., 2010). Finally, the low and high ES group differed in their neural responses to the neutral stimuli in the absence of objective flavor information.

Secondly, the relationship between the neural processing of flavor stimuli and personality traits was investigated by contrasting groups characterized by ES trait score that were either below the average range (<25th percentile) or above the average range (>75th percentile), while sample size of the groups was relatively small. Therefore, it also would be important to replicate the same relationship by using ES as a continuous predictor in a larger sample in further studies. Nevertheless, it needs to be noted that estimated power of the statistical analysis, homogeneity of variance among the groups, the use of a random effect approach, and 95% confidence intervals of the group neural responses (indicating narrow and non-overlapping ranges among the groups) suggested that differences in neural responses between the low and high ES group may reflect accurate and reliable estimations. Finally, the reported interaction effects concerning differential neural as well as behavioral responses in the ES groups were replicated by *post-hoc* analyses applied separately on each of these groups.

Thirdly, concerning the Five Factor Model of Personality, the low and high ES group also specifically differed with respect to the personality trait of Emotional Stability (but not the other dimensions) as measured by the BFQ and representing the opposite facet of neuroticism (Caprara et al., 1993). This is not surprising, since ES represents a lower order temperamental trait related to the neuroticism personality trait (Caprara and Pastorelli, 1989; Caprara et al., 1994) and is in accordance with a similar trait pattern reported by Iaria et al. (2008). Our data provide more specific information regarding the modulation by personality traits of anterior insula function, in particular, by characterizing participants on ES trait in addition to measures of more general personality traits, like Emotional Stability/Neuroticism. In other words, by putting individual differences in brain function in a more narrow context of certain facets of neuroticism that are more specifically linked with emotional responses to external stimuli, like ES trait, greater specificity can be obtained in elucidating the relationship between temperamental and personality traits and the neural processing of affect (Caprara et al., 1985; Caspi et al., 2005).

Fourthly, the relationship between individual differences in ES trait and neural responses to flavor stimuli was detected exclusively in left anterior insula. This finding differs from the relationship between ES trait and the processing of affective stimuli in bilateral anterior insula reported by Iaria et al. (2008). A principal difference between the studies regards the use of visual stimuli (pictures with affective valence) by Iaria et al. (2008) and the use of flavor stimuli (orally administered liquids with affective valence) in the present study. Although some evidence suggests bilateral involvement in the processing of distaste (Phillips et al., 1997; Jabbi et al., 2007, 2008; Stark et al., 2007), the results are consistent with other studies indicating a left hemisphere lateralization of both disgust and pleasant taste processing in anterior insula (Zald and Pardo, 2000; Royet et al., 2003; Hennenlotter et al., 2004; Dalenberg et al., 2015), even when distasting stimuli were perceived with less intensity than pleasant stimuli (Small et al., 2003). This left hemisphere anterior insula involvement in affective flavor perception is backed up by lesion studies on taste processing (Pritchard et al., 1999; Calder et al., 2000; Cereda et al., 2002; Mathy et al., 2003).

Fifthly, our participant group consisted exclusively of females. Females may differ from males in taste processing (Haase et al., 2011; Cornier et al., 2015; Nesil et al., 2015). In particular, they may be more inclined to experience disgust than males (Rozin et al., 2000; Schienle et al., 2002). However, at variance with this information, an absence of gender differences in emotion processing in cerebellum and other cortical areas has been reported, too (Baumann and Mattingley, 2012). Nevertheless, although recruitment selectivity had the advantage to study the relationship between ES trait and neural responses in a rather homogeneous group, in particular controlling for important confounding factors such as gender, caution is required in generalizing the findings to the population.

In conclusion, the findings show that individual differences in ES trait are associated with differential neural activity patterns in left anterior insula induced by flavor stimuli. An important question raised in the introduction is whether anterior insula activity is modulated by personality traits even if emotions are induced by primary sensory stimuli. The positive results of this study are compatible with the idea that some personality traits may have access to evolutionarily ancient affective systems. Moreover, we also demonstrate that insula-cerebellar intrinsic functional connectivity may be modulated by individual differences in ES trait. The stronger connectivity observed in low ES participants may be speculatively associated with a greater predisposition to identify and to discriminate the emotional value of elementary stimuli in early processing stages. The latter could be particularly relevant for a dysfunctional regulation of emotion in stressing contexts in high ES individuals.

## Funding

The study was supported by a Grant of the Italian Ministry of Health to LP (RF-2009-1547884).

### Conflict of interest statement

The authors declare that the research was conducted in the absence of any commercial or financial relationships that could be construed as a potential conflict of interest.
